# Prevention of dental caries: A review of effective treatments

**DOI:** 10.4317/jced.52890

**Published:** 2016-12-01

**Authors:** Claudio Sicca, Elena Bobbio, Natale Quartuccio, Giovanni Nicolò, Angelina Cistaro

**Affiliations:** 1 MD, Indipendent Clinical Dentistry, Forno Canavese and Bruino, Turin, Italy; 2 Dental University of Turin, Turin, Italy; 3 MD, Nuclear Medicine Unit Department of Biomedical Sciences and of Mophologic and Functional Images, University of Messina, Italy; 4 MD, Ph, Positron Emission Tomography Centre IRMET S.p.A., Affidea, Turin, Italy, PET Pediatric AIMN InterGroup, Italy, Institute of Cognitive Sciences and Technologies, CNR, Rome, Italy

## Abstract

**Background:**

The objective of this study is to review medical and non medical treatments for prevention of caries.

**Material and Methods:**

A comprehensive literature search of the most relevant and updated published studies from 01/01/2002 through December 2015 in PubMed/MEDLINE, Embase and Scopus databases regarding the efficacy of strategies and treatments aiming to prevent the development of caries was performed selecting papers on the basis of the Evidence-based Medicine Criteria.

**Results:**

We identified thirty systematic reviews on prevention of caries. Analyzing the data the retrieved literature, performance of prevention treatments seems to be high.

**Conclusions:**

Prevention treatments may have a relevant impact on the avoiding the development of caries planning.

** Key words:**Dental caries, prevention, fluoride.

## Introduction

Dental caries is a pathologic process depending on several etiologic factors, which cause the destruction of the dental tissues and produces local and general complications (1). It is one of the most widespread diseases in the civilized populations with a prevalence of 40% at the age of seven years and 85% in seventeen year-old boys (2). However there is some evidence that that incidence in children aged five-seventeen years has decreased about 36% in the last decades and approximately 50% of children can be considered caries-free in the permanent dentition (3-5).

The objective of this study is to identify proofs of efficacy of medical and non medical treatments on prevention of caries providing a review of relevant literature published in the last fourteen years.

## Material and Methods

A comprehensive computer literature search of the PubMed/MEDLINE, Embase, Scopus and Cochrane databases was conducted using a search algorithm based on the term “dental caries” used as keyword and MeSH term (oriented in “control and prevention”) in order to find relevant published articles on methods for prevention of caries. Due the extensive time period in which the topic of caries was discussed in scientific literature, 01/01/2008 was used as beginning date limit; the search was updated until December 2015. Only articles in English language were selected.

-Study Selection Criteria And Data Extraction

Only those studies that satisfied all of the following criteria were included in the present review: 1) systematic reviews performed by renowned scientific institutions; 2) acceptable quality of the studies on the basis of the standards “Consort statement”; (6) 3) only articles regarding prevention of caries from an individual and communitarian point of view were included; 4) publication date from 01/01/2002 until December 2015.

**Figure 1:**
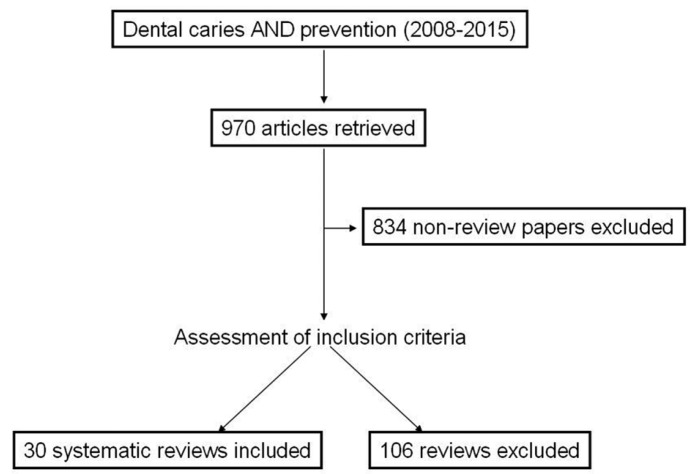
Flow-chart of study-selection.

Four researchers independently reviewed the titles and abstracts of the retrieved articles, applying the selection criteria mentioned above. Articles were rejected if they were clearly ineligible. If update versions of the paper were found, earlier versions were rejected. The same four researchers then independently reviewed the full-text version of the remaining articles to determine their eligibility for the inclusion (Fig. 1).

## Results

Using the selection criteria the computer literature search from the PubMed/MEDLINE, Embase, and Scopus revealed thirty systematic reviews (see  Table 1) (7-36). These studies were retrieved in their full-text version. No additional studies were found screening the references. The characteristics of the studies included are shown in  Table 1 presenting the systematic reviews included in the study.

**Table 1:**
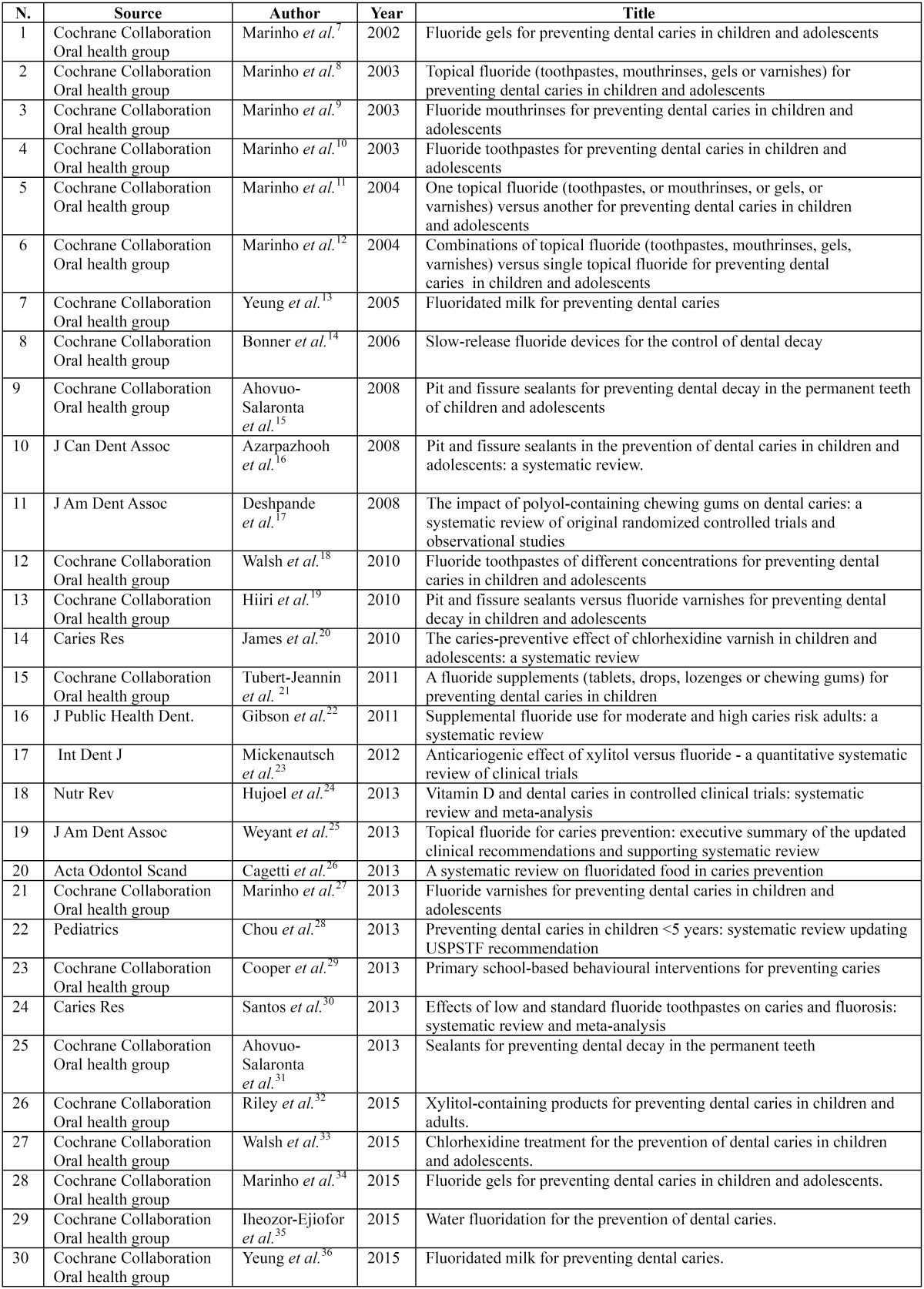
Systematic reviews included in the study.

## Discussion

Most of earlier evidences on utility of methods for prevention and control of caries come from the Cochrane group, which produced several systematic reviews on this matter so far (7-15,18,19,21,27,29,31-36).

-Fluoride gel, toothpaste and mouthrinses

In 2002, the Cochrane group evidenced that topical application of fluoride gel is associated with a substantial reduction in caries. Similarly, the same group a year later demonstrated that the use of topical systems (without distinction between toothpaste, mouthwash gels and varnishes) for fluoride therapy is clearly associated to the reduction of caries of children (8) and supervised regular use (daily or weekly) of fluoride rinse by children is associated with a clear reduction of caries (a reduction of 26% of the DMFS). This result improves by increasing the intensity of treatment (9). There is an improvement of the DMFS index correlated to the content of fluoride, the increase in the frequency of its use and the supervised brushing (10). In 2003, Marinho (11) suggested that the use of toothpaste and mouthwash compared to the use of fluoride toothpaste and fluoride gel appear to exert a similar effect in the prevention of caries in children. As no substantial differences appear between the use of paints and gels it seems to be more appropriate to use the paint because it leads to a lower intake of fluoride, however, there is insufficient evidence to define the side effects of the two interventions. The combined use of topical fluorides associated with the use of fluoride toothpaste reduces caries by at least 10%, however, there is no evidence of significant differences between the use of toothpaste and only the association of more fluoride (12). Ten years later, Weyant *et al.* (25) evaluated the efficacy of several topical fluoride agents for preventing dental caries carrying out a systematic review. The panel recommended the following fluoride agents for individuals at risk of developing decays: 2.26 % fluoride varnish or 1.23 % fluoride ( APF) gel, or a prescription-strength, home-use 0.5 % fluoride gel or paste or 0.09 % fluoride mouthrinse for six-years patients or older. Only 2.26 % fluoride varnish was recommended for children younger than six years because of their low risk of experiencing harm. The article underlined the importance of considering the patient’s risk of experiencing disease when developing a caries-prevention plan in order to balance the potential benefits and harms. According to the review, the authors suggested the use of topical fluoride agents to patients at high risk of developing dental decays considering also practitioner’s professional judgment (25).

In 2010, Walsh (18) and colleagues assessed the efficacy of fluoride toothpastes of different concentrations in preventing dental caries performing a systematic review. The purpose of the study was to compare fluoride toothpaste with placebo or fluoride toothpaste of a different concentration. They found out that there was a statistically significant discrepancy between higher fluoride toothpaste concentrations compared to placebo in preventing dental decays. As a matter of fact, the increase of fluoride concentration from 1000/1055/1250 parts per million (ppm) to 240/2500/2800 ppm improved the prevention of caries from 23% to 36% respectively compared to placebo. Contrariwise, concentrations of 440/500/550 ppm and below were not sufficient to reach a statistically significant effect. The authors confirmed the advantages of using tooth fluoride with a concentration of at least 1000ppm (18). In 2013, Santos *et al.* (30) developed a systematic review of clinical trials and meta-analysis in order to study the potential effect of low (< 600 ppm) and standard (1,000-1,500 ppm) fluoride toothpastes in preventing dental caries in the primary dentition. The authors also assessed the risk of aesthetically objectionable fluorosis in the upper anterior permanent teeth. The study demonstrated that low fluoride toothpastes significantly increased the risk of caries in the primary teeth [ RR= 1.3 (1.07-1.20)] and did not significantly decrease the risk od aesthetically fluorosis in the permanent teeth [ RR=0.32 (0.03-2.97)]. Although, it has been showed that standard fluoride toothpastes could prevent caries development, at the same time it could be a potential cause of dental fluorosis (30).

Gibson (22) and collaborators conducted a literary research about evaluating a self-or professionally applied fluoride intervention in moderate to high caries risk adults performing a randomized clinical trial. The review selected seventeen studies and findings were categorized into different groups such as NaF and amine/potassium fluoride mouthrinses of varying strengths, NaF gels and pastes, NaF varnish and stannous fluoride. Although the authors were not able to define the best dental supply because of variations in design, conduct and quality scores of the reviewed clinical trials; it was seen that low dose daily NaF rinses had the most generalizable results [RRR for carious lesions: 50-148%]. However, all demonstrated some effectiveness in preventing and/or remineralizing dental caries. According to their analysis, it was evident that more future clinical trials were required to confirm the reduction or remineralization of decays using fluoride supplies for high caries risk adults (22). In 2012, Mickenautsch *et al.* (23) performed a systematic review about the effectiveness of xylitol in comparison with the use of topical fluoride in preventing dental caries. Due to the high clinical heterogeneity, they did not perform a meta-analysis. Only caries-related primary outcomes were tested and chewing gum was the main form of clinical application in test and control groups. According to this review, the authors revealed that the use of xylitol to existing fluoride regimes could be helpful in preventing caries, however they showed a cautious conclusion because the evidence contained a high risk of bias and could be limited by confusing effects (23)

In 2015, Marinho *et al.* evaluated the performance of gels in the prevention of caries in adolescents and children. The confirmed the previous findings published in their previous systematic reviews. Indeed they documented a moderate quality evidence for fluoride gel in the inhibition of large caries in the permanent dentition (34).

The superiority of fluoride toothpaste containing xylitol over fluoride-only toothpaste for preventing caries was not demonstrated neither in a subsequent systematic review assessing this comparison in the permanent teeth of children (32).

Another systematic review compared the effectiveness of chlorhexidine against a placebo or no treatment in the prevention of caries in children and adolescents but found negligible evidence (33).

-Pit and fissure sealants 

In 2008, Ahovuo-Salaronta (15) and colleagues evaluated the caries prevention of pit and fissure sealants in children and adolescents performing systematic reviews including sixteen studies. They found significant higher benefit of second or third generation resin-based sealants on first permanent molars, compared to a control without sealant. Further, one of the studies included in the review with nine years of follow up revealed more caries in the control group compared to resin sealant group; 27% of sealed surfaces were decayed compared to 77% of surfaces without sealant. The results of the studies comparing different sealant materials were conflicting. The authors concluded that sealing is an effective method to prevent caries of the occlusal surfaces of permanent molars. According to their analysis, at high caries risk the effectiveness of sealants is clear, however there is lack of information regarding the benefits of sealing in patients with different caries risks (15). In 2008, Azarpazhooh (16) and collaborators detected 303 articles by the literature research about the effectiveness of pit and fissure sealants in preventing dental caries. The authors agreed about the application of dental sealants on all permanent molar teeth of high-risk populations without any decays and within four years after their eruption. Besides, the review showed off that resin-based sealants should had been preferred to glass ionomer cements sealants. In conclusion, their analysis underlined the importance of dental sealing without omitting the influence of fluoride varnish, education, nutritional and regular clinical consultation (16).

In 2013, Ahovuo-Saloranta *et al.* (31) analyzed the comparison between applying pit and fissure sealants versus no treatment and among different types of sealant materials for preventing dental caries in children and adolescents younger than twenty years. The resinbased sealant compared with no treatment prevented caries in the first permanent molars [ at two years of follow-up OR=0.12 ( 95% CI 0.70-0.19)] and it was revealed their effectiveness for the nine years of follow-up. On the other hand, no evidence could be issued regarding the potential prevalence among the different types of sealants because long follow-up time trials were needed. According to this review, the authors recommended the application of sealants on the occlusal surfaces of permanent molars in high-risk children to prevent and control decays (31).

-Varnishes

In 2010, Hiiri (19) and colleagues aimed to compare the efficacy of pit and fissure sealants and fluoride varnishes in order to prevent dental caries performing a systematic review. The objective was to liken also the effectiveness of sealant and varnish combination with varnish alone. They considered to treat the first molar occlusal surfaces of under twenty-year-aged children. Two studies showed a significant difference in favor of the sealants compared to fluoride varnish. In conclusion, the review supposed some evidence about the superiority sealant over varnish application in the prevention of dental decays (19). In 2010, James (20) and collaborators included in their systematic review randomised or quasi-randomised trials comparing chlorhexidine varnish to placebo, no treatment or fluoride varnish in preventing dental caries. The search included only trials on children and adolescents aged eighteen years or younger. The results were conflicting as eight trials reported no statistically significant difference in caries development in permanent teeth with the application of chlorhexidine varnish compared to placebo or no treatment. Whereas, two of them related results in favor of chlorhexidine varnish. Finally, the last two trials proved that the outcome of chlorhexidine varnish in primary teeth and the comparison among chlorhexidine varnish and fluoride varnish were dubious. The conclusions of this research were in agreement with the systematic review by Twetman confirming the evidence for a caries-preventive effect of chlorhexidine varnish was inconclusive for children and adolescents with daily exposure to fluoride (20).

In 2013, Marinho (27) and collaborators aimed to evaluate the role of professionally-applied fluoride varnishes in order to decrease dental caries in children and adolescents compared to placebo and no treatment. The trials assessed that the use of fluoride varnish on the permanent dentition was associated with a 43% ( 95% IC 30% to 57%) reduction in DMFT; whereas on the primary dentition with a 37% ( 95% IC 24% to 51%) reduction. According to the outcomes of this review, the authors found that the application of fluoride varnishes two or four times a year was associated with a significant decrease of decays in population with different levels of caries risk and exposure to other relevant details such as potential side effects and those related to acceptability of treatment (27).

-Fluoridated supplements, water and milk.

Chou *et al.* (28) assessed the efficacy of preventing dental caries in children younger than five years of age including preventive treatments such as dietary fluoride supplementation, fluoride varnish and xylitol. The authors updated the 2004 US Preventing Services Task Force recommendation. The review showed no new randomized trials about the effectiveness of dietary fluoride supplementation. Newer observational studies regarded potential association between early childhood intake of fluoride supplementation and risk of enamel fluorosis, which was usually mild. Evidence remained limited even on the use of xylitol in children aged five and younger. Lastly, evidence was found in using fluoride varnish in order to reduce caries incidence in high-risk children. According to this, three new randomized trials were consistent in finding fluoride varnish more effective than no varnish as a 18% to 59% caries reduction. In conclusion, the authors suggest the necessity of further researches about the efficacy of the screening and the preventive interventions by primary care providers in early childhood (28). Instead, Cooper (29) and collaborators performed a systematic review evaluating the influence of behavioral interventions delivered in primary school children for preventing dental caries including the changing behavior related to toothbrushing habits and the frequency of consumption of cariogenic food and drinks. Studies were generally less than two years in length and showed lack of uniformity in describing the interventions; no meta-analysis could be conducted on caries outcomes, neither on plaque outcomes because of high heterogeneity.

Hujoel (24) and colleagues assessed the impact of vitamin D for dental caries prevention performing a systematic review selecting twenty-nine references from the literature. The relative-rate estimates of the twenty-four CCTs showed significant heterogeneity (*P* < 0.0001) and there was evidence of significant publication bias (*P* < 0.001). The systematic review revealed that supplemental vitamin D was associated with a 53% (95% CI, 43%-65%) reduced risk of caries. The authors did not identified robust differences among the effectiveness of UV therapy and nutritional supplementation with either vitamin D2 or vitamin D3. In summary, the review suggested the usage of vitamin D in early life, before the age of thirteen years, in order to prevent dental caries, however further researches should be carried out (24).

In 2008, Deshpande and Jadad (17) dealt with the importance of polyol-containing chewing gums in preventing dental caries performing a meta-analysis. The study was carried out on about 8,600 school-aged children and it focused on comparisons between polyol-containing chewing gum and no chewing gum as control. The results showed a statistically significance of using xylitol, xylitol-sorbitol blend and sorbitol in preventing caries as they revealed a PF ( prevented fraction) of 58.66 % (35.42-81.90), 52.82 % (39.64-66.00) and 20.01 percent (12.74-27.27), respectively. Instead, the combination of sorbitol and mannitol-containing chewing gum was not associated with difference in ΔDMFS when compared with no chewing gum [( PF of 10.71%(?20.50-41.93)]. The authors suggested the use of polyols as part of normal oral hygiene thanks to their effect on *S. mutans* and on salivary dynamics triggered by chewing process. However, the systematic review concluded underlining that the number and designs of the studies were not sufficient to draw firm findings around dose-response relationships and the relative effectiveness of different polyols (17). In 2011, Tubert-Jeannin (21) and colleagues assessed the key role of fluoride supplements for preventing dental decays in 7196 schoolchildren. The main aim of the review was to evaluate whether there were differential effects between fluoride supplements in the form of tablets, drops, lozenges and chewing gums and no fluoride supplements or other preventing measures such as topical fluorides. On permanent dentition, the results suggested that there was a 24% ( 95% confidence interval 16% to 33%) reduction on average in decayed, missing and filled tooth surfaces with the use of fluoride supplements compared to the control group.

In 2015 another study reported on the effect of water fluoridation in the prevention of caries. The authors stated that most of the available data derived from studies conducted prior to 1975 suggest the effectiveness of water fluoridation against caries levels in children. However these findings may be hamperd by the observational nature of the study designs and the different lifestyle of our age compared to the seventies. Thus there is not sufficient evidence to determine whether the use water fluoridation has a significant impact in the reduction of caries (35).

In 2013, Cagetti *et al.* (26) conducted a systematic review regarding the effectiveness of fluoridated food in caries prevention, excluding water. The assessments recorded studies of the effect of fluoridated milk in children primary teeth and the results un-derlined the improvement in preventing decays. In Jamaica a work was conducted about the impact of salt fluoridation among children and the authors concluded that a reduction in caries prevalence can be obtained, but without any scientific evidences. According to this review, the efficacy of food fluoridation in preventing dental decays was low and the majority of the trials have been conducted in children (26).

Also the fluoridate milk, despite the poor quality of the studies retrieved in the systematic review of Yeung (13), would seem to provide benefits to children in school age, especially for the permanent dentition. The use of slow-release fluoride devices would seem to be a protection system, however, the generalizability of this practice appears difficult and of questionable applicability given the poor practicality of the devices (14). Finally Yeung *et al.* produced another review on fluoridated milk confirming the results of his previous systematic review but highlighting the low quality of evidence of the utility of adding fluoridated milk in the diet of schoolchildren (36).

Conclusions

Prevention treatments may have a relevant impact on the avoiding the development of caries. On the basis of this large review, a greater diffusion of preventive interventions could reduce the incidence of caries at an affordable cost. Topical application of fluoride gel and fluoride supplements appear convenient and inexpensive tools to reduce dental caries. Likely, also pit and fissure sealants and fluoridate varnishes appear to effectively reduce the risk of caries.

Food fluoridation, fluoridated milk and fluoridated water do not seem, based on the existing literature, to hold sufficient evidence for the reduction of dental caries.
